# Pronounced differences in heart rate and metabolism distinguish daily torpor and short-term hibernation in two bat species

**DOI:** 10.1038/s41598-022-25590-8

**Published:** 2022-12-15

**Authors:** Shannon E. Currie, Gerhard Körtner, Fritz Geiser

**Affiliations:** 1 Centre for Behavioural and Physiological Ecology, Zoology CO2, University of New England, Armidale, NSW 2351 Australia; 2 Functional Ecology, Institute of Zoology, Universität Hamburg, Hamburg, Germany

**Keywords:** Ecology, Physiology, Zoology

## Abstract

Torpor, and its differential expression, is essential to the survival of many mammals and birds. Physiological characteristics of torpor appear to vary between those species that express strict daily heterothermy and those capable of multiday hibernation, but comparisons are complicated by the temperature-dependence of variables. Previous reviews have compared these different torpor strategies by measuring the depth and duration of torpor in multiple species. However, direct comparison of multiple physiological parameters under similar thermal conditions are lacking. Here, we quantified three physiological variables; body temperature, metabolic rate (MR) and heart rate (HR) of two small heterothermic bats (daily heterotherm *Syconycteris australis,* and hibernator *Nyctophilus gouldi*) under comparable thermal conditions and torpor bout durations. When normothermic and resting both MR and HR were similar for the two species. However, during torpor the minimum HR was more than fivefold higher, and minimum MR was 6.5-fold higher for the daily heterotherm than for the hibernator at the same subcutaneous T_b_ (16 ± 0.5 °C). The data show that the degree of heterothermy defined using T_b_ is not necessarily a precise proxy for physiological capacity during torpor in these bats and is unlikely to reveal accurate energy budgets. Our study provides evidence supporting a distinction between daily torpor in a daily heterotherm and short term torpor in a hibernator, at least within the Chiroptera with regard to these physiological variables. This exists even when individuals display the same degree of T_b_ reduction, which has clear implications for the modelling of their energy expenditure.

## Introduction

Torpor expression has commonly been classified into two major strategies; daily torpor seen in daily heterotherms and multiday torpor seen in hibernators^1^. The comparison between these groups is complicated, however, by the strong and often direct temperature-dependence of variables of torpor, leading some to conclude that heterothermy falls on a body temperature (T_b_) dependent continuum e.g.^2^. Yet there is a lack of consistent data that scrutinize the difference between these strategies under similar thermal conditions^3^. A proper understanding of the differences between daily heterotherms and hibernators in their physiological capacity for torpor use is essential for understanding the flexibility of their physiology and behaviour, particularly in our rapidly changing world.

Traditional interpretations of physiological capacity in heterotherms are derived from data on species that exhibit the extremes in torpor pattern and have become ‘model’ species of thermal biology, such as obligate seasonal hibernators^4,5^ or strict daily heterotherms^6,7^. Yet evidence is amassing from non-Holarctic species, and the wild, that indicates flexibility in torpor use across species and throughout the year^8,9^. It is evident that multiday torpor is not restricted to winter^10^, and species capable of multiday torpor can also enter short bouts of torpor throughout the year^11^. For example, the little brown bat *Myotis lucifugus* is capable of torpor bouts up to 45 days during winter hibernation, but in summer regularly expresses torpor bouts lasting less than 1 day^12^. Yet these short bouts are often referred to as ‘daily torpor’^13^ due to their temporal resemblance to torpor in daily heterotherms. Furthermore these bouts often occur at high ambient temperatures (T_a_), where the degree of T_b_ reduction may also be similar among species. For example, the mouse lemur *Microcebus griseorufus,* is capable of multiday torpor bouts at a skin temperature of around 20 °C, but also can exhibit short torpor bouts of only a few hours in the same season^14^, making it difficult to distinguish from a daily heterotherm using either T_b_ or torpor bout duration alone.

The maximum degree of metabolic rate reduction has been used to distinguish daily heterotherms from hibernators in > 200 species^15^. This distinction is thought to be driven by the fact that in daily heterotherms metabolism during torpor is, to a large extent, a function of body temperature^16^, whereas hibernators possess the ability to further inhibit metabolism physiologically^17^. If daily heterotherms and hibernators differ in their degree of metabolic rate reduction, even at the same body temperature, then the short bouts of torpor in hibernators will provide greater energy savings than daily torpor in daily heterotherms. Altogether, this highlights the importance of considering ‘non-model’ species, conducting experiments under a range of ambient conditions, and including more proximal physiological traits in investigations^9^.

Bats are an ideal group in which to study torpor as they have an almost global distribution and the vast majority of species studied have been shown to express torpor^18^; even in the tropics, subtropics and arid zones^19–22^. Torpor strategies in bats are highly flexible. Unlike other Holarctic hibernating species, many bats are able to enter short bouts of deep torpor throughout the year and to enter multiday torpor, at high ambient temperatures^23,24^. While most research on torpor in bats has focussed on species that enter multiday torpor, detailed knowledge about daily heterothermy is scant. This is particularly true for measures of heart rate, with only a single record from a pteropodid in torpor^25^.

Here we compared heart rate (HR), oxygen consumption ($\dot{\rm V}{\text{O}_{2}}$; a proxy for metabolism) and subcutaneous temperature (T_sub_) throughout torpor and at rest for two bat species that express either daily heterothermy (the 18 g blossom bat, *Syconycteris australis,* Pteropodidae), or hibernation (the 10 g long-eared bat, *Nyctophilus gouldi*, Vespertilionidae) to assess whether short-term torpor in a hibernator is comparable to a daily heterotherm. *Syconycteris australis* exhibit torpor patterns indicative of a daily heterotherm with torpor bouts generally < 12 h and minimum T_b_ in torpor of 18 °C^26,27^. In addition, this species has one of the highest recorded field metabolic rates for endotherms, suggesting that while torpor is essential for energy balance, the savings are minimal in this species as they only enter short, shallow torpor bouts in the wild^28^. *Nyctophilus gouldi*, while capable of hibernation, also enters short bouts of torpor throughout the year^29^. Previous investigations have shown that there is no effect of season on the electrocardiogram in this species during torpor^30^, which suggests that short torpor bouts may not differ physiologically from multiday torpor bouts at the same temperature. These two species overlap in their geographic distributions, providing a unique opportunity to compare hibernator and daily heterotherm species exposed to similar environment conditions in the wild and therefore experiencing similar selection pressures for torpor use. We hypothesized that the degree of energy savings from short torpor bouts in the hibernator would differ from the daily heterotherm, and that this would be evident even when species were exposed to the same ambient conditions. Furthermore, we predicted that cardiac function and metabolism would be lower in the hibernator than the daily heterotherm.

## Results

### Oxygen consumption and heart rate at rest

**Figure 1:**
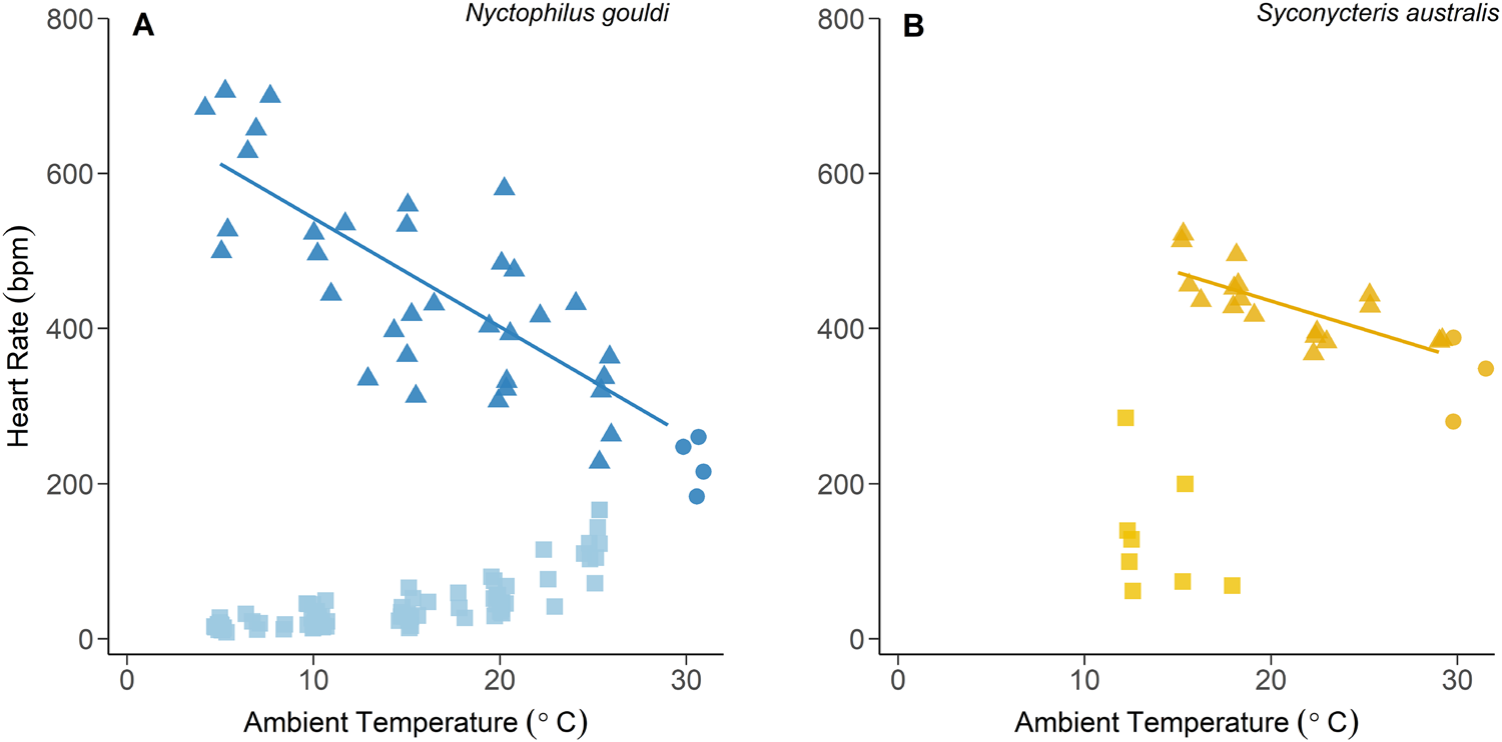
Heart rate as a function of ambient temperature (T_a_) for *Nyctophilus gouldi* and *Syconycteris australis*. Basal HR (filled circles) was recorded in the thermoneutral zone (TNZ; 29.5–34 °C). Below the TNZ, resting HR (triangles) increased linearly for both species; (**A**) HR (bpm) = 682.57 -14.03 (T_a_), (**B**) HR (bpm) = 582.32 -7.34 (T_a_). Bats entered torpor (filled squares) when exposed to T_a_ below 25 °C (*Ng*) and 20 °C (*Sa*).

When bats were normothermic (*S. australis, n* = 4, T_sub_ = 33.8 ± 0.9 °C; *N. gouldi*, *n* = 13, T_sub_ = 34.4 ± 1.2 °C) $\dot{\rm V}{\text{O}_{2}}$ increased linearly with decreasing T_a_ below the thermoneutral zone (Supplementary Figure 1A & B). Although $\dot{\rm V}{\text{O}_{2}}$ appeared to increase more steeply in *N. gouldi* than *S. australis*, reflecting the difference in size and hence thermal conductance of the species, this difference in slope was not found to be statistically significant (lme, *df* = 25.09, t = 1.99, *p* = 0.06). This was also the case for resting HR (lme, *df* = 37.02, t = 1.70, *p* = 0.10). For *N. gouldi,* resting HR increased by 182 bpm between T_a_ 25 °C and 12 °C (Fig. 1A). In contrast, for *S. australis* the change in resting HR (95 bpm) was just over half that of *N. gouldi* over the same temperature range (Fig. 1B).

**Figure 2:**
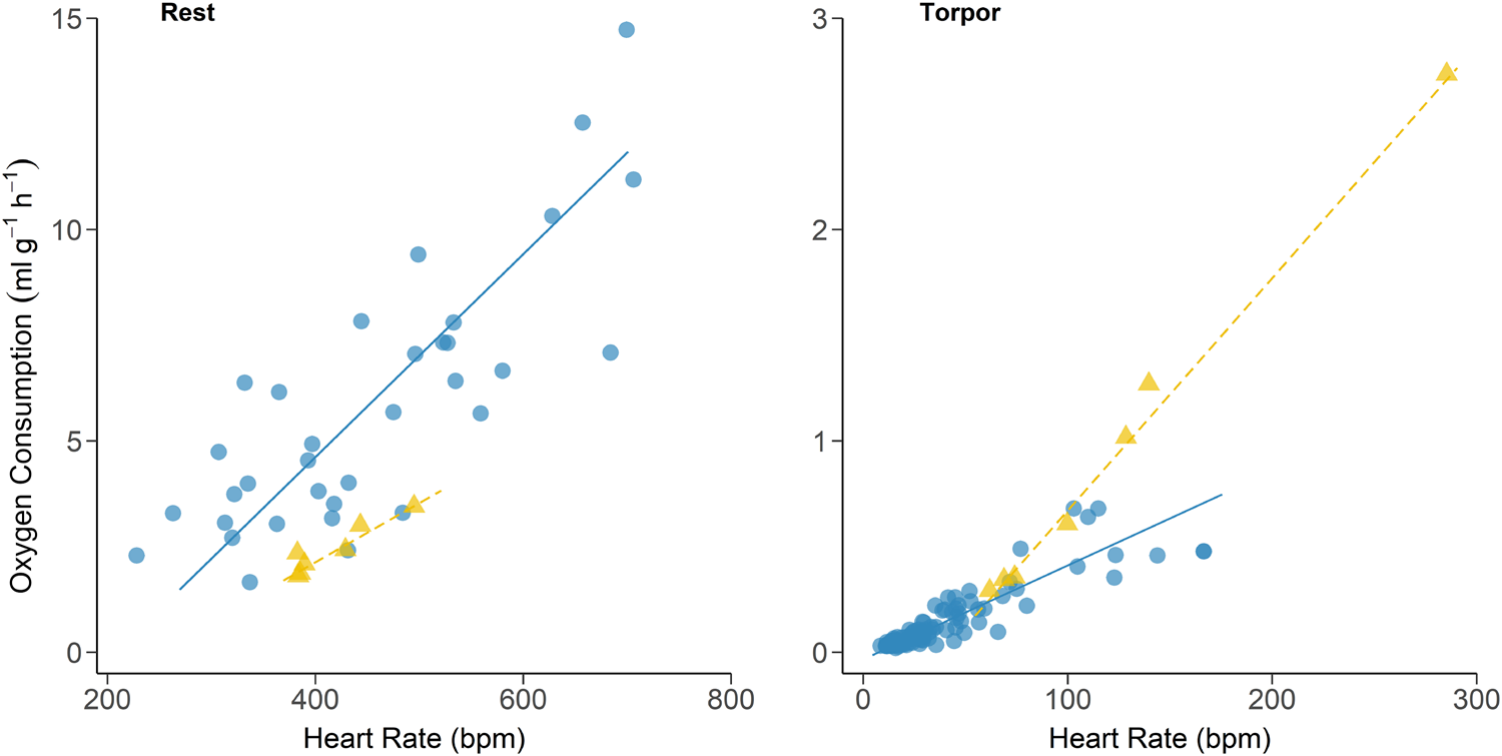
The relationship between HR and $\dot{\rm V}{\text{O}_{2}}$ for *N. gouldi* (blue circles-solid line) and *S. australis* (yellow triangles-dashed line) during rest and torpor. *N. gouldi*; resting $\dot{\rm V}{\text{O}_{2}}$ = 0.024(HR) − 5.104, r^2^ = 0.65, *p* < 0.001, torpid $\dot{\rm V}{\text{O}_{2}}$ = 0.004(HR) − 0.034, r^2^ = 0.78, *p* < 0.001. *S. australis*; resting $\dot{\rm V}{\text{O}_{2}}$ = 0.014(HR) − 3.467309, r^2^ = 0.88, *p* < 0.01, torpid $\dot{\rm V}{\text{O}_{2}}$ = 0.011(HR) − 0.429, r^2^ = 0.99, *p* < 0.001.

When we compare the relationship between resting $\dot{\rm V}{\text{O}_{2}}$ and resting HR, however, the differences between the species became more pronounced (Fig. 2; sma; *df* = 1, χ = 5.32, *p* < 0.05). For every 10 bpm increase in HR the rate of increase in $\dot{\rm V}{\text{O}_{2}}$ was almost twice as high for *N. gouldi* than *S. australis*; increasing by 0.24 ml O_2_ g^−1^ h^−1^ versus 0.14 ml O_2_ g^−1^ h^−1^ respectively.

Consequently, in *N. gouldi* oxygen pulse increased from an average of 1.01 × 10^−4^ ml O_2_ g^−1^ beat^−1^ when metabolic rate was basal in the thermoneutral zone, to an estimated 2.35 × 10^−4^ ml O_2_ g^−1^ beat^−1^ at T_a_ 12 °C (data taken from the curve fitted to the data). This represents a 48.7% increase in HR to account for increased $\dot{\rm V}{\text{O}_{2}}$, whereas for *S. australis* oxygen pulse increased from 0.89 × 10^−4^ to 1.57 × 10^−4^ ml O_2_ g^−1^ beat^−1^, which equated to a contribution of HR of only 38.3%.

### Heart rate and metabolism during torpor

When bats were resting at similar T_a_ (15.0 ± 0.7 °C) both resting HR and $\dot{\rm V}{\text{O}_{2}}$ were comparable between the species, with only a 51 bpm difference in resting HR and less than 0.5 ml g^−1^ h^−1^ difference in $\dot{\rm V}{\text{O}_{2}}$ (Table 1). However, when both species entered torpor minimum HR and $\dot{\rm V}{\text{O}_{2}}$ were more than fourfold higher on average in *S. australis* than *N. gouldi* (Table 1). When we compared individuals at their thermoconforming minimum, when T_sub_ was within 1 °C of one another, the difference in $\dot{\rm V}{\text{O}_{2}}$ was even more pronounced at ninefold higher in *S. australis* than *N. gouldi* (0.36 versus 0.04 ml O_2_ g^−1^ h^−1^ respectively). In these same individuals heart rate was more than fivefold higher in *S. australis* than *N. gouldi* (74 and 14 bpm respectively). This difference was also found for the distributions of torpid HR and $\dot{\rm V}{\text{O}_{2}}$ over the same T_a_ range (Fig. 2; ks-test for HR, D = 0.86, *p* < 0.001; ks-test for $\dot{\rm V}{\text{O}_{2}}$, D = 0.93, *p* < 0.001).

**Table 1:** Average physiological parameters for *Syconycteris australis* and *Nyctophilus gouldi* at rest (normothermia) and in torpor where ambient temperature was 15 ± 0.7 °C (range 13–16.5 °C).

	*Syconycteris australis*	*Nyctophilus gouldi*
*HR* (bpm;* rest*)	482 ± 42 (*n = 3, N* = *4*)	431 ± 88 (*n = 7, N* = *7*)
*HR* (bpm; *torpor*)	137 ± 89 (*n* = *2, N = 2*)	32 ± 12 (*n = 17, N* = *19*)
$\dot{V}{O}_{2}$ (ml g^−1^ h^−1^; *rest*)	4.38 ± 0.28 (*n = 4. N* = *4*)	4.79 ± 1.91 (*n* = *7, N = 7*)
$\dot{V}{O}_{2}$ (ml g^−1^ h^−1^; *torpor*)	0.7 ± 0.55 (*n = 2, N* = *5*)	0.1 ± 0.04 (*n = 17, N* = *19*)
*T*_*sub*_ (°C; *rest*)	34.0 ± 0.9 (*n=4, N* = *8*)	34.4 ± 1.5 (*n = 6, N* = *6*)
*T*_*sub*_ (°C; *torpor*)	19.9 ± 3.5 (*n = 4, N* = *6*)	16.0 ± 0.8 (*n = 17, N* = 20)

The proportional reduction of $\dot{\rm V}{\text{O}_{2}}$ during torpor was greater in *N. gouldi* than *S. australis* at the same T_a_ (15.1 ± 0.1 °C). Average $\dot{\rm V}{\text{O}_{2}}$ during torpor was 7.4 ± 0.7% of basal metabolic rate in *N. gouldi* compared to 31.7 ± 9.5% for *S. australis*. This was also true of the reduction in HR proportionate to basal heart rate, which was only 14.0 ± 1.2% in *N. gouldi* individuals compared to 60.7 ± 11.8% for *S. australis.* If we consider this in relation to resting metabolic rate at the same temperature, *N. gouldi* showed a 97.9 ± 0.2% reduction in $\dot{\rm V}{\text{O}_{2}}$ during torpor compared to an average 88.4 ± 3.5% reduction for *S. australis*. Again, the proportional reduction in heart rate was also greater in *N. gouldi* than *S. australis* when compared to resting heart rate at the same T_a_ (92.6 ± 0.6% versus 71.6 ± 8.3%).

The absolute minimum HR in torpor for *S. australis* was 69 bpm recorded at T_a_ 12.6 °C with T_sub_ of 15.0 °C. The corresponding minimum $\dot{\rm V}{\text{O}_{2}}$ was 0.29 ml g^−1^ h^−1^. The minimum HR in torpid *N. gouldii* was less than one quarter of *S. australis* at a similar temperature (T_a_ = 10.7 °C, T_sub_ = 11.2 °C) at 16 bpm and $\dot{\rm V}{\text{O}_{2}}$ was one tenth that of *S. australis* at 0.02 ml g^−1^ h^−1^. Yet the absolute minimum HR we recorded in *N. gouldi* fell as low as 9 bpm (T_sub_ = 5.8 °C) with corresponding $\dot{\rm V}{\text{O}_{2}}$ of 0.03 ml g^−1^ h^−1^ as these individuals were exposed to lower T_a_ than *S. australis* (in this case 5 °C).

During steady-state torpor bats of both species showed strong linear relationships between HR and $\dot{\rm V}{\text{O}_{2}}$ (*Sa* r^2^ = 0.99, *Ng* r^2^ = 0.78, *p* < 0.01) (Fig. 2). However, there was a significant difference between the relationships for each species (χ = 23.38, *df* = 1, *p* < 0.001) as $\dot{\rm V}{\text{O}_{2}}$ increased more steeply with increasing HR in torpid *S. australis* than for *N. gouldi*.

### Subcutaneous temperature during torpor

**Figure 3:**
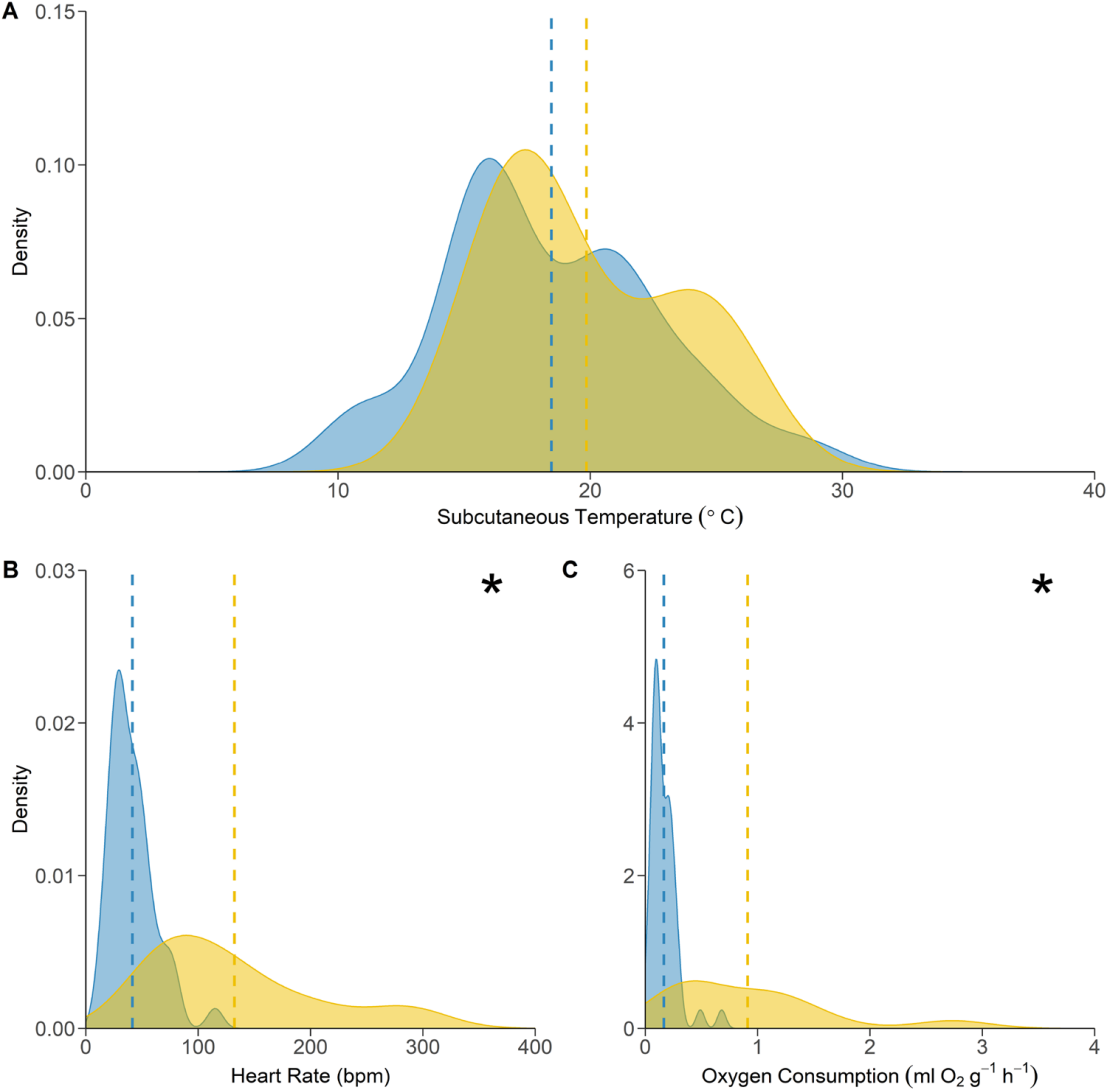
Kernel-density distributions of physiological parameters in torpor for *N. gouldi* (blue shading) and *S. australis* (yellow shading) at ambient temperatures between 10.5 and 24 °C. Mean values represented by dashed vertical lines. The distributions of both heart rate and oxygen consumption in torpor differed significantly between the two species (as determined via Kolmogorov–Smirnov tests and shown with asterix) even though subcutaneous temperature in torpor was not different.

Both bat species maintained a mean T_sub_ around 34 °C (*Sa* 33.8 ± 0.9 °C; *Ng* 34.4 ± 1.2 °C) when normothermic and resting (Supplementary Figure 2). When animals entered torpor the relationship between T_sub_ and T_a_ differed between the two species, even though the distribution of T_sub_ of torpid bats over the same T_a_ range did not (Fig. 3; ks-test, D = 0.30, *p* = 0.55). *Nyctophilus gouldi* entered torpor at T_a_ ≤ 25 °C and T_sub_ remained within 2 °C of T_a_ in thermoconforming torpid individuals. Only eight individuals maintained T_sub_ > 2 °C (5.1 ± 0.7 °C) above T_a_ and these bats did so at mild temperatures between 19 and 22 °C on only one occasion each. In contrast, *S. australis* maintained a greater differential between T_sub_ and T_a_ when in torpor below 20 °C (5.4 ± 3.9 °C on average) and T_sub_ during torpor was highly variable at any given T_a_. For example, T_sub_ ranged from 16.3 to 24.1 °C at T_a_ 14.9 ± 0.9 °C. Yet, the minimum T_sub_ for *S. australis* was 15.0 °C recorded at T_a_ of 12.6 °C.

### Torpor entry and arousal

**Figure 4:**
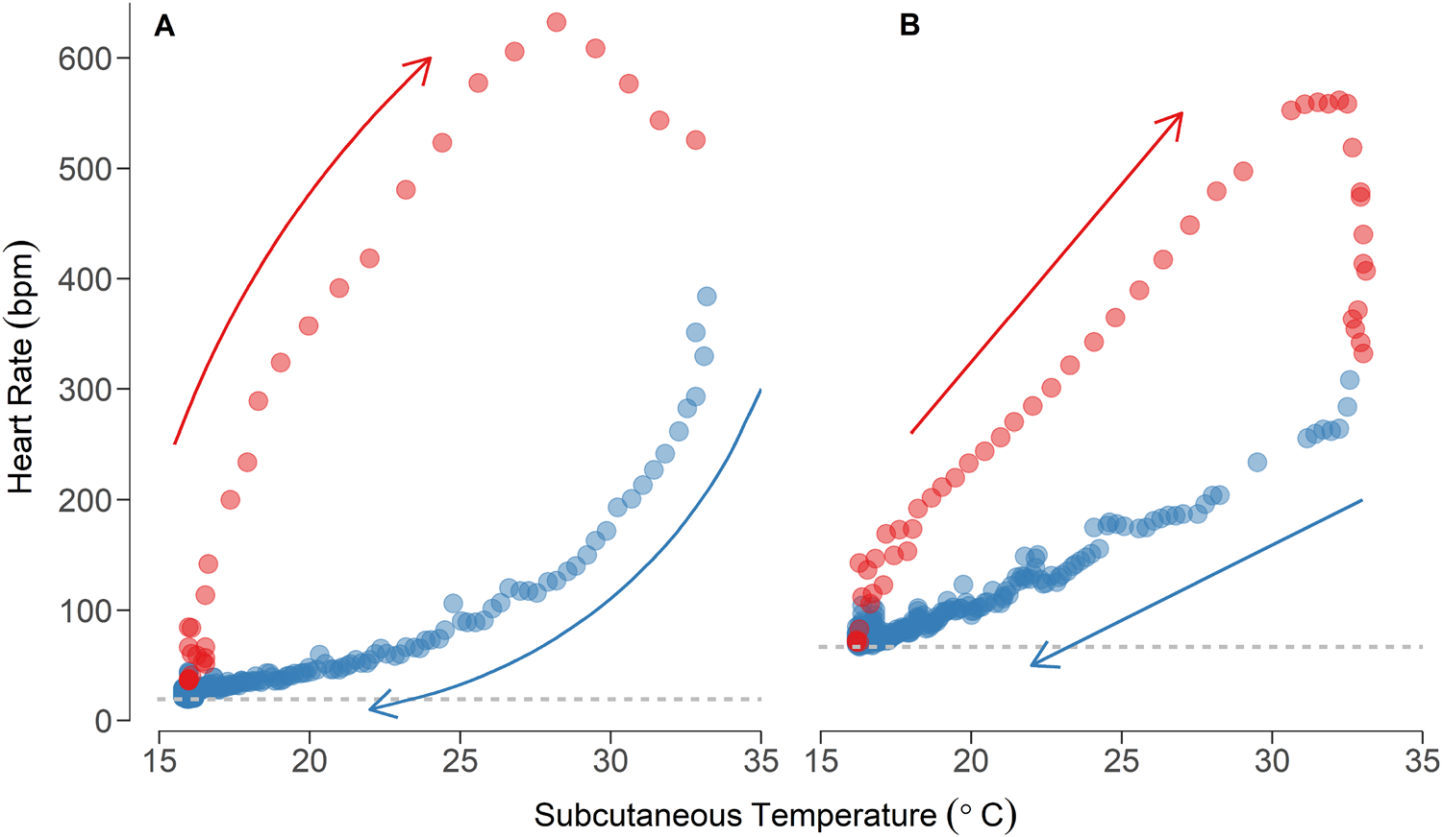
Heart rate as a function of subcutaneous temperature during entry (blue circles and arrow) and arousal (red circles and arrow) from a single torpor bout for *N. gouldi* (**A**) and *S. australis *(**B**) at an ambient temperature of 15 °C. Arrows indicated the progression of the torpor bout in time and dashed lines indicate minimum heart rate during torpor.

HR showed a pronounced hysteresis with T_sub_ during entry and arousal from torpor in both species, although absolute values of HR differed substantially between the two species (Fig. 4). During entry into torpor HR was slower at each T_sub_ than at the corresponding T_sub_ during rewarming. Figure 4 also illustrates the pronounced drop in HR during torpor entry with little change in T_sub_ in *N. gouldi* compared to a shallower linear decline in HR in *S. australis*. Yet the rapid increase in HR during rewarming over a small T_sub_ change and overshoot of HR at the end of arousal from torpor was similar in both species. For example, at the initiation of arousal in *N. gouldi* HR increased ~ eightfold over a rise in T_sub_ of only 1.6 °C, from 24 to 200 bpm. Similarly, HR of *S. australis* more than doubled when rewarming over a T_sub_ range of only 1.4 °C from 70 to 173 bpm. The overall time taken to rewarm showed a negative curvilinear response to increasing T_a_ (Supplementary Figure 3). The log transformed data showed a significant relationship between time taken to rewarm and T_a_ for *N. gouldi* (lme, *df* = 46.1, t = − 11.715, *p* < 0.01). Yet post-hoc analysis revealed no effect of T_a_ on arousal time for *S. australis* (lme, *df* = 35.15, t = − 0.741, *p* = 0.464) and no overall difference between the species (lme, *df* = 35.73, t = 0.631, *p* = 0.532).

## Discussion

Although HR, $\dot{\rm V}{\text{O}_{2}}$ and T_sub_ were similar in resting normothermic bats at mild T_a_, when bats entered torpor the mechanisms reducing HR, $\dot{\rm V}{\text{O}_{2}}$ and T_sub_ were decidedly different between the daily heterotherm and hibernator. At all T_a_ measured, the hibernator *N. gouldi*, had lower $\dot{\rm V}{\text{O}_{2}}$ and HR during torpor than corresponding measurements for the daily heterotherm, *S. australis*. This was true even when there was no significant difference in the distribution of T_sub_ between the two groups.

Our results are consistent when compared across several bat species whose geographic ranges overlap with *N. gouldi* and/or *S. australis*. During short term torpor at 15 °C, metabolic rate in two species capable of hibernation (10 g *Nyctophilus bifax* and 15 g *Chalinolobus gouldii*)*,* was less than one third of *S. australis* at the same T_a_; 0.11 and 0.06 versus 0.36 ml O_2_ g^−1^ h^−1^ respectively^31,32^. Heart rate was also substantially lower in torpid *C. gouldii* at only 20 bpm compared to our findings of 74 bpm for *S. australis* when T_b_ was ~ 16 °C^32^. This is interesting considering that these data were taken from *N. bifax* captured at the same location as *S. australis* in this study^31^, and that the range of body mass in *C. gouldii* converges with *S. australis*^33^. When we compare this to data from the only other daily heterotherm within this geographic area where torpor has been reported (16 g *Macroglossus minimus*) minimum metabolic rate in torpor was similar to that of *S. australis* at the same T_a_; 0.58 versus 0.60 ml O_2_ g^−1^ h^−1^ at 18 °C^26,34^. These minima are more than fivefold the recorded metabolic rate of *C. gouldii* and threefold our findings for *N. gouldi* at the same T_a_ (0.10 ± 0.04 and 0.17 ± 0.06 ml O_2_ g^−1^ h^−1^, respectively)^32^. Taken together this suggests that the degree of physiological reduction in daily heterotherms and hibernators may be consistently different across bat species, even at the same ambient temperatures. It is important to recognise that these comparisons are between bats from two different families (Vespertilionidae and Pteropodidae), which complicates our comparison as the distinctions between torpor patterns are also divided across phylogenetic lines^15^. Further investigation of more species at the same ambient conditions is necessary for us to clarify these distinctions.

While our sample size for *S. australis* may be low, our findings for metabolic rate during torpor are supported by those of previous studies on this species which report mean minima between 0.47 and 0.75 ml O_2_ g^−1^ h^−1^^27,34,35^.The only other pteropodid bat for which HR has been measured during torpor is the daily heterotherm *Nyctimene albiventer* (28 g) and even then, only two recordings were made^25^. During torpor at T_a_ 25 °C HR of *N. albiventer* was relatively low at 88 and 96 bpm with torpid $\dot{\rm V}{\text{O}_{2}}$ of 0.67 ± 0.17 ml O_2_ g^−1^ h^−1^. Minimum average HR recorded for *S. australis* in our study was only slightly lower, 62 bpm at T_a_ 12.6 °C which corresponded to a $\dot{\rm V}{\text{O}_{2}}$ of 0.29 ml O_2_ g^−1^ h^−1^, less than half that of *N. albiventer*. While we are confident that our data accurately reflect the cardiac function of *S. australis* in torpor and at rest, our small sample size should be taken into consideration. Therefore, we advocate that more investigation into cardiac function in daily heterotherm species, including *S. australis*, be undertaken to improve our understanding of this important physiological variable.

Short term torpor in bat species capable of hibernation is often referred to as daily torpor suggesting that it may differ from seasonal multiday torpor^36^. This is partially due to the fact that short bouts often occur at mild T_a_, but may also reflect the assumption that hibernation requires seasonal preparation; such as tissue remodelling and differential gene expression^37^. Data reported for hibernating bat species after many days in torpor show that HR falls to around 10 bpm at T_a_ 5 °C^38,39^. Our data for *N. gouldi* are comparable, if not lower than those presented during longer torpor bouts at the same T_a_, confirming that short term torpor in these bats is physiologically indistinguishable from multiday hibernation. This also supports previous findings for this species that showed no seasonal difference in electrocardiogram parameters during torpor^30^. The capacity for energy savings in hibernators even during short torpor bouts is important to recognise and investigate as there is a rise in the number of studies modelling energy expenditure in bats under varied ecological and climate change scenarios^40,41^.

Entry into torpor progressed more slowly in the daily heterotherm than the hibernator, reflected by a slower decline in HR during cooling, as illustrated by a more gradual slope of the hysteresis curve in *S. australis* compared to *N. gouldi* at the same T_a_. During entry into torpor the hypothalamic set point for thermoregulation gradually falls to the new low set point for steady-state torpor^42^. Our results suggest that the fall in T_set_ occurs more slowly in daily heterotherms as animals defend T_b_ throughout, or for part of, cooling. The progression of torpor entry in the hibernator was more rapid, likely as a function of metabolic inhibition and active suppression of HR at the onset of cooling, and the much lower T_set_ in torpor.

The ability to rewarm from torpor is a defining feature of heterothermic animals and the arousal process is extremely costly. Arousal costs are reduced when rewarming rate is maximised^43^ and the importance of this was evident in both species of bats investigated here as there was no significant effect of torpor pattern on the time taken to rewarm. Our data for HR during rewarming also showed that both species were capable of increasing HR at the onset of arousal to ~ 200 bpm before T_sub_ had increased by more than 2 °C. This indicates that selection for swift rewarming may be consistent across species regardless of the depth or length of torpor bouts, as has been previously suggested^44^.

During periods of normothermy both bat species showed a qualitatively similar response of HR and $\dot{\rm V}{\text{O}_{2}}$ to changes in T_a_ following the general endothermic pattern. Our results demonstrate that the relationship between HR and $\dot{\rm V}{\text{O}_{2}}$ at rest vary between the two species reflected in both regression analyses and calculations of oxygen pulse. Although the oxygen pulse of both species increased with decreasing T_a_, the percentage contribution of HR to oxygen transport differed between the two species. The proportional increase in HR for increased oxygen transport at low T_a_ was substantially lower in *S. australis* (38.3%) than *N. gouldi* (48.7%) across an equivalent T_a_ gradient. This suggests that for *S. australis* other aspects of the cardiovascular system, such as stroke volume, play a greater role in maintaining cardiac output at high metabolic rate. It is possible that these differences relate to energy acquisition and partitioning associated with flight and may not necessarily be a function of the species’ pattern of torpor use. Nectar feeding bats and birds are known to have the highest maximum rate of oxygen consumption amongst animals and consequently nectarivorous bats have comparatively high field metabolic rates^28,45^. Furthermore, flight dynamics have been shown to be highly correlated with heart mass^46^. It is possible that the differences reported here for resting HR and the relationship with $\dot{\rm V}{\text{O}_{2}}$ in *N. gouldi* and *S. australis* relate to some extent to the relative size of the heart^46^ and a greater cardiac reserve which might be necessary for longer foraging times in the nectar feeding bats. However, this relationship should be reversed during torpor, as the larger mass and heart size in *Syconycteris* should result in a lower HR than in the smaller *Nyctophilus*, emphasising the physiological differences between these groups in relation to torpor.

From an ecological perspective, an understanding of the differences in physiological capacity between daily heterotherms and hibernators gives us a stronger tool to model and predict how environmental conditions effect energy budgets for these species. Here we show that a similar reduction in body temperature does not equate to the same level of energy savings for two species of heterothermic bat, and that these differences persist across a number of bat species at the same T_a_. Ideally, with more data, phylogenetically controlled analyses could be incorporated to better address whether these differences reflect physiologically distinct groups (daily heterotherms vs hibernators), or are simply a function of phylogenetic effects (vespertilionids vs pteropodids). If short-term torpor of the same duration exhibited by *N. gouldi* was mistaken for daily torpor and ascribed a similar proportional reduction in metabolism to *S. australis* (8% of resting metabolism in *S. australis* compared to 0.8% for *N. gouldi* at the same T_sub_) we would substantially overestimate the daily energy needs of these individuals. Using a novel approach of investigating different torpor patterns under similar experimental conditions, we provide evidence that short term torpor in vespertilionid hibernators provides proportionately greater energy savings than daily torpor in pteropodid daily heterotherms. Moreover, we provide additional evidence for the use of heart rate as a mechanism to investigate physiological capacity in heterothermic species.

## Methods

### Study species

*Syconycteris australis* are nectarivorous bats from tropical and subtropical eastern Australia and in captivity have been shown to use daily torpor throughout the year. Torpor bouts are generally restricted to < 12 h and minimum regulated T_b_ reported during torpor is ~ 18 °C^27,35^. The propensity of these bats to enter torpor has been suggested to have enabled *S. australis* to extend its range into higher latitudes where it overlaps with temperate species such as *N. gouldi* (Supplementary Figure 4)^47,48^. *Nyctophilus gouldi* are insectivorous bats that hibernate in winter, expressing torpor bouts of around 2 weeks and often express short-term torpor bouts of < 24 h throughout the year^29^. These bats maintain a low T_b_ during both short and long torpor bouts with reported minimum regulated T_b_ of ~ 1–2 °C^49^.

Adult male *S. australis* (*n* = 4, body mass at capture = 18.7 ± 0.5 g) were caught in mist nets at Iluka Nature Reserve in a subtropical habitat on the north coast of NSW, Australia (29°24’S, 153°22’E). Bats were transported to the University of New England (UNE) and housed in a large indoor flight cage (2 × 2 × 2 m), which was equipped with branches and large stands of foliage for individuals to roost in. Temperature of the room was maintained at 20–22 °C, with relative humidity > 55% and animals were exposed to natural light. Food, a blended mixture of fruit, juice and protein supplements^27^ was available ad libitum in modified plastic syringes that acted as feeders which were placed among branches. Feeders were refilled daily and washed/soaked overnight in Milton antibacterial solution to minimize microbial growth. Water was available in birdfeeders.

Adult *N. gouldi* (*n* = 21, body mass at capture = 10.5 ± 0.3 g) were caught at Imbota Nature Reserve and Newholme Field Station in a temperate habitat near Armidale, NSW (30°35’S, 151°44’E). At UNE, bats were kept in large outdoor flight cages (3 × 1.5 × 2 m) with a maximum of eight animals per cage and provided mealworms and water ad libitum. Mealworms were dusted with a supplement of Wombaroo™ Insectivore Rearing Mix twice a week. Additional food was supplied in the form of moths and other flying insects, and these were attracted into cages by a UV light. All bats were kept in captivity for a maximum period of seven months and each individual remained within 1 g of their body mass at the time of capture while in captivity (average BM; *S. australis* = 19.6 ± 0.7 g, *N. gouldi* = 11.0 ± 0.3 g).

### Animal ethics declaration

This study was conducted under a scientific license provided by the NSW Parks and Wildlife Authority (SL100084) under the guidelines of the Environment Protection and Biodiversity Conservation Act 1999*.* Animal Ethics approval was granted by the animal ethics committee of the University of New England (AEC11-016 and AEC12-043). This study was carried out in compliance with the ARRIVE guidelines on animal research and the Australian Code for the Care and Use of Animals for Scientific Purposes*.*

### Subcutaneous body temperature

Temperature-sensitive transponders (IPTT-300 Bio Medic Data Systems, Delaware, USA) were used to measure subcutaneous body temperature (T_sub_). Transponders were calibrated over a range of 5 to 40 °C to the nearest 0.1 °C against a precision reference thermometer in a water bath prior to use. Bats were given a minimum of 3 days to acclimate to captivity and ensure stable body mass before transponder implantation. Transponders were implanted interscapularly under general Isoflurane/oxygen anaesthesia. The skin was cleaned with 70% alcohol before a small (~ 3 mm) incision was made in the skin just below the shoulder blades for insertion. The insertion site was closed with a single suture (chromic gut, Ethicon, Somerville, MA, USA). Bats were given 24 h to recover in small individual cages in a warm room before being returned to their flight cages.

### Respirometry

Individuals were placed in respirometry chambers in the late afternoon/early evening and oxygen consumption ($\dot{\rm V}{\text{O}_{2}}$) was measured as a proxy for MR over the course of the following day (at least ~ 20 h). Bats were weighed (to 0.1 g) before the start of experimentation and immediately after being removed from respirometry chambers. We calculated mass-specific $\dot{\rm V}{\text{O}_{2}}$ assuming a linear rate of mass loss. Bats were exposed to T_a_ within the typical range of their natural habitat, as such the *N. gouldi* were measured from T_a_ 33 °C down to 4 °C and *S. australis* were only measured from T_a_ 33 °C down to 12 °C. During measurements bats were exposed to a simulated natural photoperiod.

Oxygen concentration of excurrent air was measured on a FC-1B Oxygen Analyser (Sable Systems, Nevada, USA). Measurements were taken from airflow through the chamber every minute for 15 min and then switched to outside air for reference readings (3 min). Chamber T_a_ was measured to the nearest 0.1 °C using a calibrated thermocouple placed ~ 5 mm within the chamber. Outputs of the digital thermocouple thermometer, flowmeter and oxygen analyser were recorded onto a personal computer using custom written data acquisition software (Gerhard Körtner) and $\dot{\rm V}{\text{O}_{2}}$ was calculated using equation 3A of Withers^50^. T_sub_ was read from each animal with a DAS-7006/7R/S Handheld Reader (Bio Medic Data Systems, Delaware, USA) which was connected to a personal computer and programmed to take readings every minute, concurrent with respirometry measurements.

As body mass and roosting posture differed between the two species, respirometry chambers were of different sizes and roosting material was also different. *Nyctophilus gouldi* were placed in rectangular polycarbonate chambers (0.26, 0.40, or 0.53 l) where they roosted flush with the back wall of the chamber and clung to a small patch of hessian cloth. *Syconycteris australis* preferred to hang in the centre of the chamber (glass jar 0.75 l) where they were free hanging and not touching the glass. Individuals roosted from plastic mesh supported horizontally near the roof of the chamber by a wire frame. Flow rate was adjusted (180–290 ml min^−1^) to ensure that 99% equilibrium was reached in < 15 min. $\dot{\rm V}{\text{O}_{2}}$ measurements were time adjusted for lag of the system, but not washout characteristics of the chambers, to correspond with measurements of HR and T_sub_.

#### Electrocardiogram (ECG) measurements

For both species of bat ECGs could only be measured during the day as the animals did not tolerate electrode wires during their active phase (overnight). Following lights on in the morning, most bats were either already torpid or had begun to enter torpor (depending on T_a_ and species) and at that point ECG electrode wires were attached to the bats’ forearms (lead I arrangement). This disturbance resulted in a partial arousal and bats either remained normothermic and resting for the remainder of recording or returned to torpor, either with or without ECG electrodes attached. *S. australis* individuals were less likely to return to torpor, hence our smaller sample size for this species. However, metabolic rate in torpor was unchanged, or even slightly lower, in both species following ECG lead attachment (paired t-test; *p* = 0.07 *Nyctophilus,* *p* = 0.20 *Syconycteris*), therefore the data we were able to collect reflect undisturbed torpor.

For *N. gouldi* ECG’s were recorded following the methods of Currie^30^ using adhesive electrodes of appropriate length and width to fit the forearm. Adhesive electrodes were not suitable for the forearms of *S. australis* however, and thus we constructed ECG electrodes from modified metal ear tags for mice that fit around the forearm of the bats in a similar fashion to bat ID bands. Bands were kept on the animals throughout time spent in captivity and only one bat experienced irritation associated with the bands. In this case the band was removed and the individual excluded from experimentation until completed healed. Electrode leads were made from modified Kittycat™ Paediatric Monitoring Electrodes (Tyco Healthcare Group, Mansfield, USA) with stainless steel clips at one end. ECG electrode gel was applied to the metal clips to improve signal conduction only for *S. australis*.

### Statistical analysis

To objectively assess whether the physiology of torpor differs between the two species, analyses were restricted to short torpor bouts of less than 24 h. Minimum values of T_sub_, HR, and $\dot{\rm V}{\text{O}_{2}}$ were averaged over 30 min from animals in steady-state torpor. On occasion transponders temporarily stopped working when the T_sub_ of *N. gouldi* in torpor fell below 7 °C. In these cases T_sub_ was estimated to be 0.5 °C above T_a_, as this was the average differential for animals with similar $\dot{\rm V}{\text{O}_{2}}$ whose transponders continued to work at low T_a_.

Resting VO_2_ and HR for both species were either taken from animals that did not enter torpor during the day, or from the period following arousal. Additional resting VO_2_ values for *S. australis* were taken from the period in the afternoon when animals were first placed in the respirometry chamber. Basal metabolic rate (BMR) and basal heart rate (BHR) were measured following arousal from torpor while animals were resting and within the thermoneutral zone previously determined for each species^27,49^. Duration of arousal was calculated from the point at which T_sub_ began a steady continuous increase until maximum T_sub_ was reached for both species (average 34.9 ± 1.5 °C, range 32.1–37.9 °C).

The percentage contribution of resting HR to increased metabolism in normothermic individuals at low T_a_, was calculated using the equation of Bartholomew and Tucker^51^;

$$\% {\text{HR}} = \frac{{HR_{2} - HR_{1} }}{{HR_{1} }} \div \left( {\frac{{HR_{2} - HR_{1} }}{{HR_{1} }} + \frac{{OP_{2} - OP_{1} }}{{OP_{1} }}} \right) \times 100$$

using HR and oxygen pulse (OP; $\dot{\rm V}{\text{O}_{2}}$/HR) at T_a_ 1 (TNZ; 30.4 ± 0.8 °C) and T_a_ 2 (12.0 °C).

Statistical analyses were performed using R v4.0.3. Linear mixed effects models were used to interpret and compare the relationship between physiological variables and T_a_ between the species using the *lme4* package with species as a fixed factor and individual as a random effect^52^. We used the package *lmerTest* to provide *p*-values for linear mixed effects models^53^. Within this package degrees of freedom and t-values are calculated using the Welch-Satterthwaite equation. Data was relevelled and tests were repeated to specifically assess the effect of T_a_ for each species, as such *p*-values were corrected using the Holm-Bonferroni method. Standardized major axis regressions were performed to assess the relationship between HR and $\dot{\rm V}{\text{O}_{2}}$ using the *smatr* package^54^ as this enables us to account for measurement errors on both the x and y axes^55^. Pseudo-replication was accounted for by using the degrees of freedom as for mixed effects modelling in *lmerTest*. To determine whether the distributions of HR, MR and T_sub_ of torpid bats form a single continuous distribution for both species under the same ambient conditions, Kolmogorov–Smirnov tests were performed for all data collected at T_a_ between 10.5 and 24 °C.

## Supplementary Information

Supplementary Information.

## Data Availability

The datasets used and/or analysed during the current study available from the corresponding author on reasonable request.
