# Supplementary material for: Pronounced differences in heart rate and metabolism distinguish daily torpor and short-term hibernation in two bat species
Source: Sci Rep. 2022 Dec 15;12:21721. doi: 10.1038/s41598-022-25590-8 (PMC9755216; doi:10.1038/s41598-022-25590-8)
Supplement: Supplementary file 1 — Supplementary Information. [file 41598_2022_25590_MOESM1_ESM.docx]

**Supplementary Material**


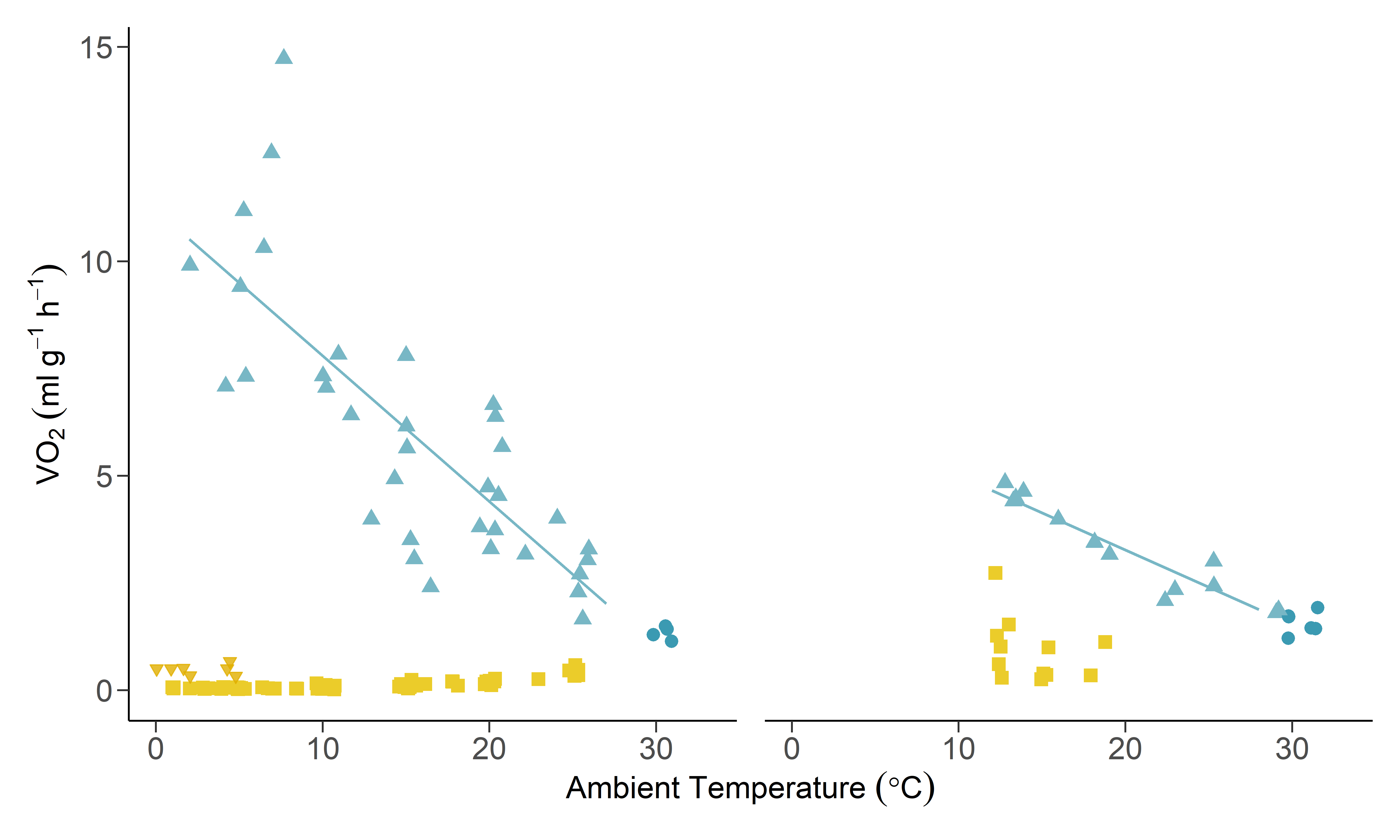


**A) B)**

**Supplementary Figure 1.** V̇O_2_ as a function of T_a_ for **A)** *Nyctophilus gouldi* and **B)** *S. australis*. BMR (filled grey circles) was recorded in the thermoneutral zone 29.5-34°C. Below this resting V̇O_2_ (circles) increased linearly; **A)** V̇O_2_ = 11.2-0.34(T_a_), r^2^=0.64, p <0.001 **B)** V̇O_2_ = 6.74-0.17(T_a_), r^2^=0.90, p<0.001. Bats entered torpor (filled yellow squares) when exposed to T_a_ below 20°C. Below T_a_ 3°C *N. gouldi* individuals began thermoregulating when torpid (grey triangles).

**Supplementary Figure 2.** Subcutaneous temperature (T_sub_) as a function of ambient temperature (T_a_) during torpor (filled circles) and at rest (circles). Solid line represents the line of equality (T_sub_=T_a_) A) *S. australis* individuals entered torpor below T_a_ of 20°C maintained a T_sub_ in torpor >15°C B) *N. gouldi* individuals entered torpor at T_a_ ≤ 25°C and thermoconformed in torpor down to ~3-5°C (depending on the individual).


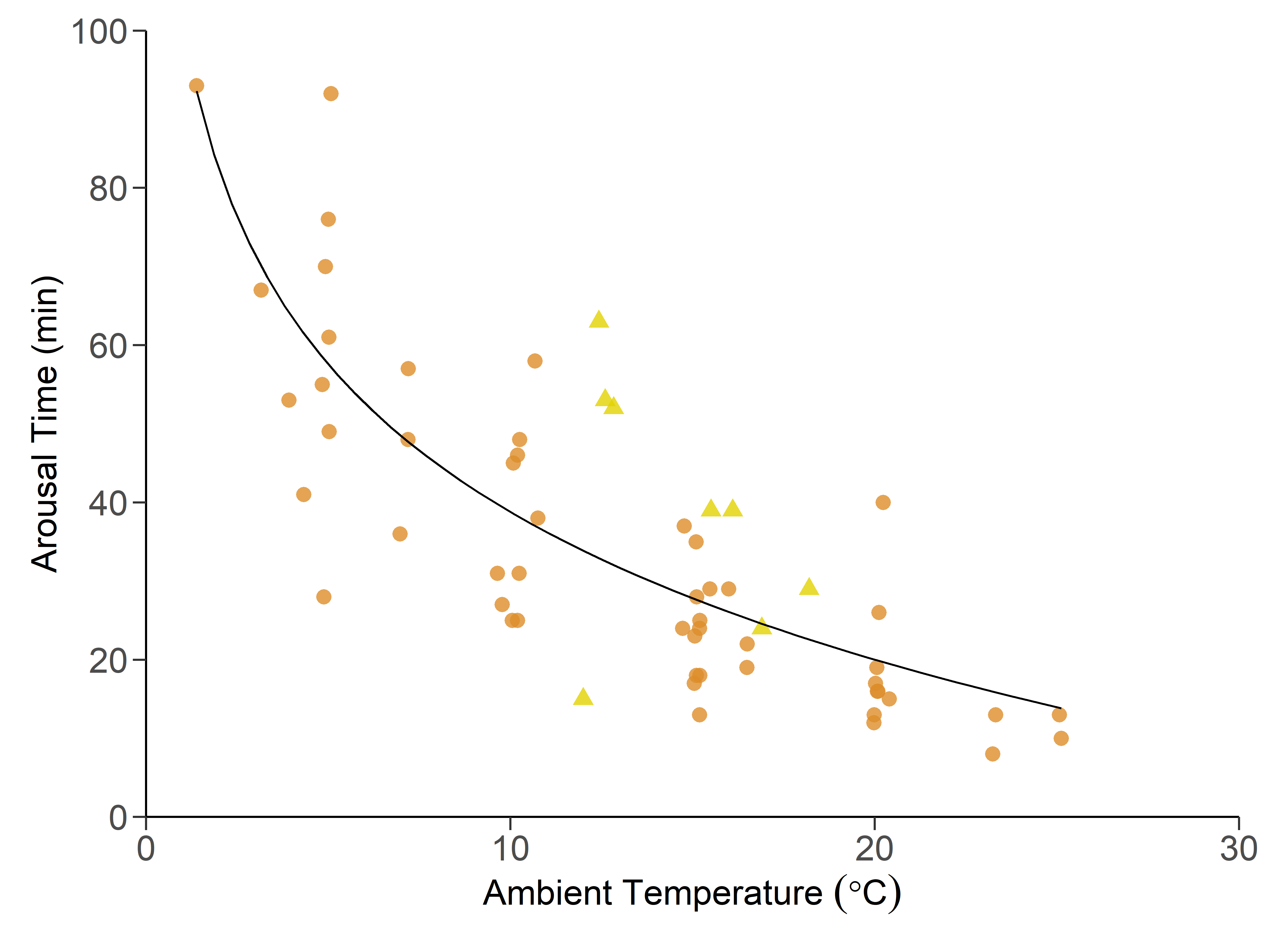


**Supplementary Figure 3.** Time taken to arouse as a function of T_a_ for both species (*N. gouldi*; filled circles, *S. australis*; circles). There was no significant effect of species and as such data were pooled.


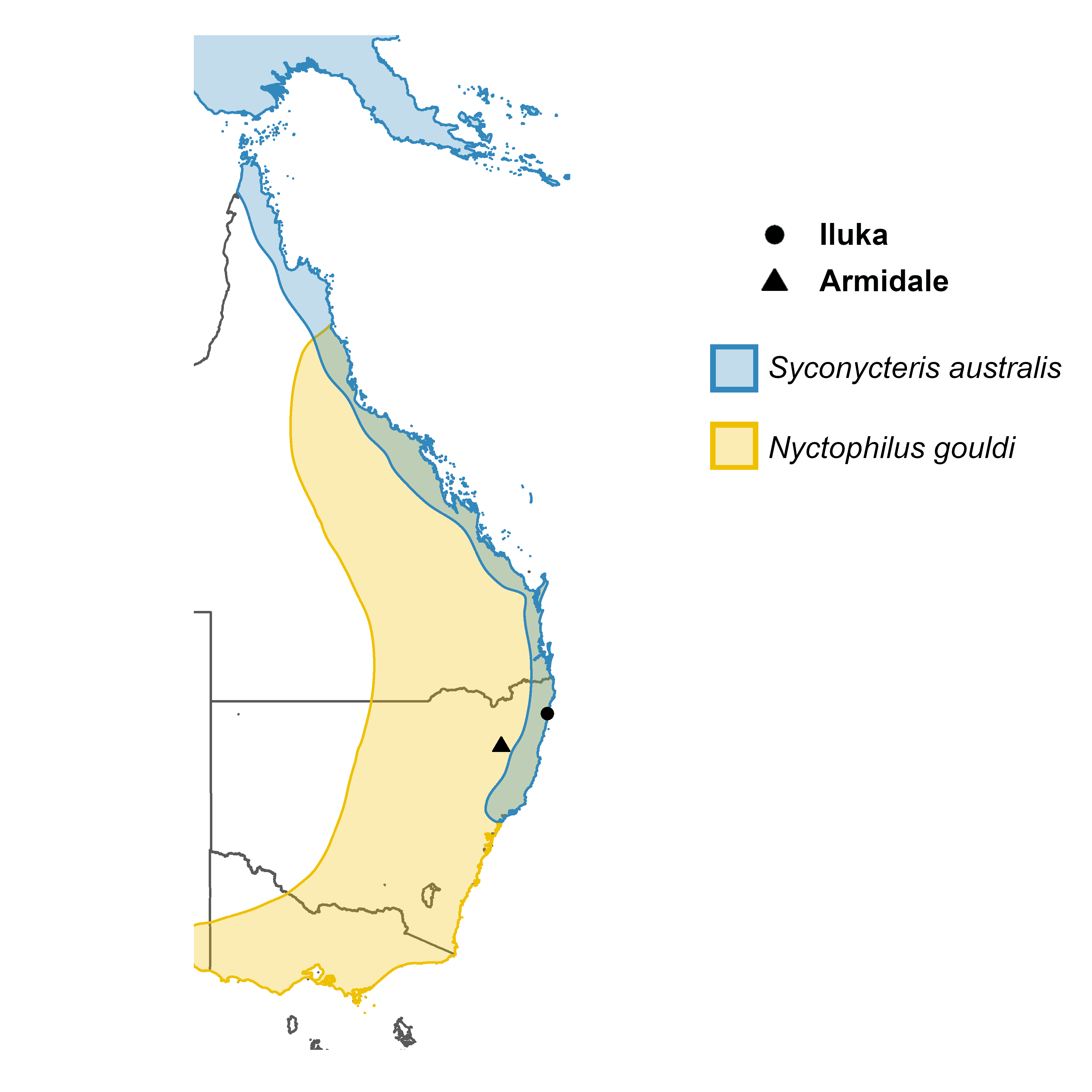


**Supplementary Figure 4.** Map of eastern Australia showing the distribution of *S. australis* (blue shading) and *N. gouldi* (yellow shading) along with the locations of their capture. Map generated using R Studio using packages *ggplot2* (Wickham, 2016) and *sf* (Pebesma, 2018) with shape files for species distribution taken from the Map of Life database (Marsh et al., 2022).

**REFERENCES**

1. **Marsh, C. J. Sica, Y. V. Burgin, C. J. Dorman, W. A. Anderson, R. C. Del Toro Mijares, I. Vigneron, J. G. Barve, V. Dombrowik, V. L. Duong, M. et al.** (2022). Expert range maps of global mammal distributions harmonised to three taxonomic authorities. *J Biogeogr* **49**, 979-992.
2. **Pebesma, E.** (2018). Simple Features for R: Standardized Support for Spatial Vecotr Data. *The R Journal* **10**, 439-446.
3. **Wickham, H.** (2016). ggplot2: Elegant Graphics for Data Analysis: Springer-Verlag New York.
